# Myasthenia gravis in 2025: five new things and four hopes for the future

**DOI:** 10.1007/s00415-025-12922-7

**Published:** 2025-02-22

**Authors:** S. N. M. Binks, I. M. Morse, Mohammad Ashraghi, A. Vincent, Patrick Waters, M Isabel Leite

**Affiliations:** 1 Nuffield Department of Clinical Neurosciences, University of Oxford, Oxford, UK; 2 Department of Neurology, John Radcliffe Hospital, Oxford, UK; 3 Medical Sciences Division, John Radcliffe Hospital, University of Oxford, Oxford, UK

**Keywords:** Clinical guidelines, Myasthenia gravis, Neuromuscular junction, Thymectomy, Older adults, Rituximab

## Abstract

The last 10 years has brought transformative developments in the effective treatment of myasthenia gravis (MG). Beginning with the randomized trial of thymectomy in myasthenia gravis that demonstrated efficacy of thymectomy in nonthymomatous MG, several new treatment approaches have completed successful clinical trials and regulatory launch. These modalities, including B cell depletion, complement inhibition, and blockade of the neonatal Fc receptor, are now in use, offering prospects of sustained remission and neuromuscular protection in what is a long-term disease. In this review, we update our clinico-immunological review of 2016 with these important advances, examine their role in treatment algorithms, and focus attention on key issues of biomarkers for prognostication and the growing cohort of older patients, both those with long-term disease, and late-onset MG (‘LOMG’). We close by expressing our four hopes for the next 5–10 years: improvements in laboratory medicine to facilitate rapid diagnosis, effective strategies for neuromuscular protection, more research into and better understanding of pathophysiology and treatment response in older individuals, and the potentially transformative role of therapies aimed at delivering a durable response such as chimeric antigen receptor (CAR) T cells. Our postscript summarizes some emerging themes in the field of serological and online biomarkers, which may develop greater stature in the next epoch.

## Introduction

In 2016, as the milestone of the randomized trial of thymectomy in generalized myasthenia gravis (gMG) approached, our review ‘Myasthenia gravis: a clinical-immunological update’ looked forward to this pivotal trial, and outlined and forecast other key developments. Since that date, the treatment landscape in MG has expanded even beyond anticipation with multiple new immunotherapies arriving in clinical practice. We continue to see an increase in late-onset MG (LOMG) and also long-standing disease in people living with MG for decades, requiring thoughtful practice and consideration of the impact of age, co-morbidities and long-term immunosuppression on these populations.

In this review, we will focus on ‘five new things’ which, in our view, constitute the most transformative developments in the field since our 2016 review, and then express ‘four hopes’ for the future across different domains of MG in the next 10 years. Our ‘five new things’ include thymectomy, new immunotherapies, recent guidelines, progress in biomarkers, and the concept of MG ‘age’ and ‘stage’, and restricting ourselves to acetylcholine receptor (AChR) antibody-positive and muscle-specific kinase (MuSK) antibody-positive patients. We will not focus in depth on ocular or seronegative MG, recapitulate established treatments, or touch on less frequently found antibodies.

## Five new things

### Thymectomy: the MGTX trial comes of age

The rationale for thymectomy in nonthymomatous MG is to remove the thymus as a key source of autoimmunization and autoantibody-secreting cells (ASCs) [1]. Since our earlier review, the randomized trial of thymectomy in myasthenia gravis (MGTX) demonstrated the therapeutic benefit of thymectomy in nonthymomatous MG and reinforced the thymus’ pivotal role in MG immunopathology [1]. Histologically examined specimens showed thymic follicular hyperplasia and atrophy, with cortical atrophy more prominent in subjects > 50 years old [1]. The primary outcomes of the trial were mean quantitative myasthenia gravis (QMG) score [2] and prednisone requirement over the three-year study duration. The group randomized to thymectomy plus prednisone had lower mean QMG scores (5.47 vs 9.34; 4 = 0.0007) and dose requirements (24 mg vs 48 mg; *p* = 0.0002) than the non-surgical group: the first study to validate the benefit of thymectomy in nonthymomatous MG. The mean reduction of 2.85 points between thymectomized and non-thymectomized groups exceeded a previously identified clinically meaningful threshold of 2.3 [3]. Throughout the initial MGTX study and its smaller two-year extension [4], the thymectomy plus prednisone group demonstrated a higher rate of achieving minimal manifestation status (MMS) (i.e. not experiencing functional limitations) while having discontinued prednisone treatment [1, 5].

In all cases of thymoma, regardless of MG status, thymectomy is crucial unless contraindicated [6].

MGTX employed an open, transsternal approach and, as anticipated, the application of minimally invasive thymectomy techniques is now common. For thymectomy, video-assisted thoracoscopic surgery (VATS) and robot-assisted thoracoscopic surgery (RATS) are widely utilized [7–9]. These methods are associated with improved peri-operative measures, including reduced blood loss, reduced pain and shorter hospital stay compared to traditional open (transsternal) thymectomy [10–12]. In the absence of prospective studies to guide decision-making, choice of thoracoscopic technique is currently dictated by regional availability and surgeons’ preference and experience [12]. There is a paucity of randomized trials to evaluate surgical techniques and none have yet been published although a single case–control study is underway (Andreas Meisel, 2021, Clinicaltrials.gov ID: NCT04158661).

Ongoing research into histological differences between patients might advance patient stratification or prognostication in the future. One digital analysis of thymic samples found a positive correlation between the number of ectopic germinal centers and post-operative improvement in nonthymomatous MG [13]. However, it is important to recognize the limitations of conventional imaging to detect thymic hyperplasia; in a retrospective study of 106 cases from our center, MRI thorax missed all such cases, whereas the sensitivity of CT thorax, while better, was still only 28.6%. Therefore, histological diagnoses should not be conferred on imaging findings alone. [14]

Around 15% of MG patients lack detectable serum AChR antibodies. Within this subgroup, immunoreactivity is directed against proteins such as muscle-specific kinase (MuSK) or low-density lipoprotein receptor-related protein 4 (LRP4) [15]. There is limited evidence supporting the efficacy of thymectomy in these patients [16]. Observational data indicate that thymectomy has a therapeutic benefit in a proportion of juvenile MG patients, but it remains to be seen whether this is sustained over extended periods [17, 18]. Finally, in ocular MG (OMG), thymectomy is only indicated in patients in whom pharmacological agents fail or are altogether contraindicated [16].

In summary, thymectomy will certainly remain a cornerstone of MG management in patients with AChR antibodies. Advances in surgical techniques, patient stratification and disease pathology will continue to influence patient outcomes. Furthermore, real-world data should provide insights into the remission rates and risk of thymoma recurrence associated with novel minimally invasive techniques. Where possible, advancements should be bolstered by evidence from prospective studies to bridge the gaps in current understanding.

### The expanding treatment landscape in myasthenia gravis

**Fig. 1:**
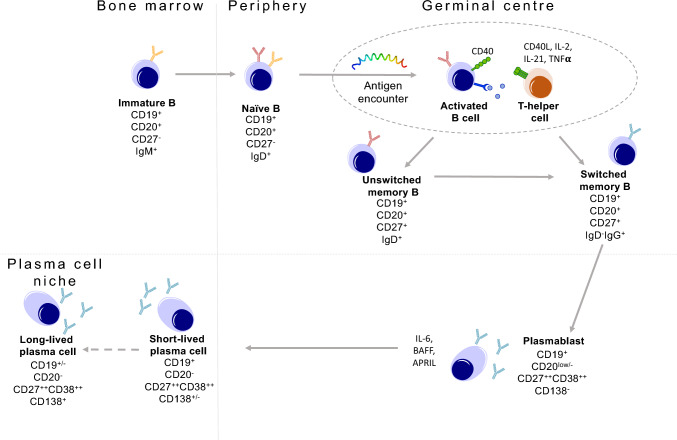
Summary of the B cell lineage differentiation and associated cell-surface phenotypes. Bone marrow emigrant naïve antigen-inexperienced B cells encounter antigen and T cells in a germinal center. Germinal centers are most commonly located in lymph nodes and spleen. The T cells express CD40L and secrete IL-2, IL-21 and TNFa, among other factors which help naïve B cells differentiate into CD27+ unswitched (IgD+) and switched (IgG+) memory B cells. Unswitched memory B cells may also express IgM. These then differentiate into antibody-secreting cells (below the dashed line: plasmablasts, short- and long-lived plasma cells) whose survival is supported by IL-6, BAFF and APRIL. Short-lived plasma cells may reside in tissues including bone marrow. Long-lived plasma cells typically niche in the bone marrow, but can reside in the central nervous system in states of inflammation. Antibodies in blue = IgG, red = IgD; yellow = IgM. Figure and caption reproduced with minor alteration from: Condition-dependent generation of aquaporin-4 antibodies from circulating B cells in neuromyelitis optica, Wilson et al. [22] This is an open access article distributed under the terms of the Creative Commons CC BY license, which permits unrestricted use, distribution, and reproduction in any medium, provided the original work is properly cited

**Fig. 2:**
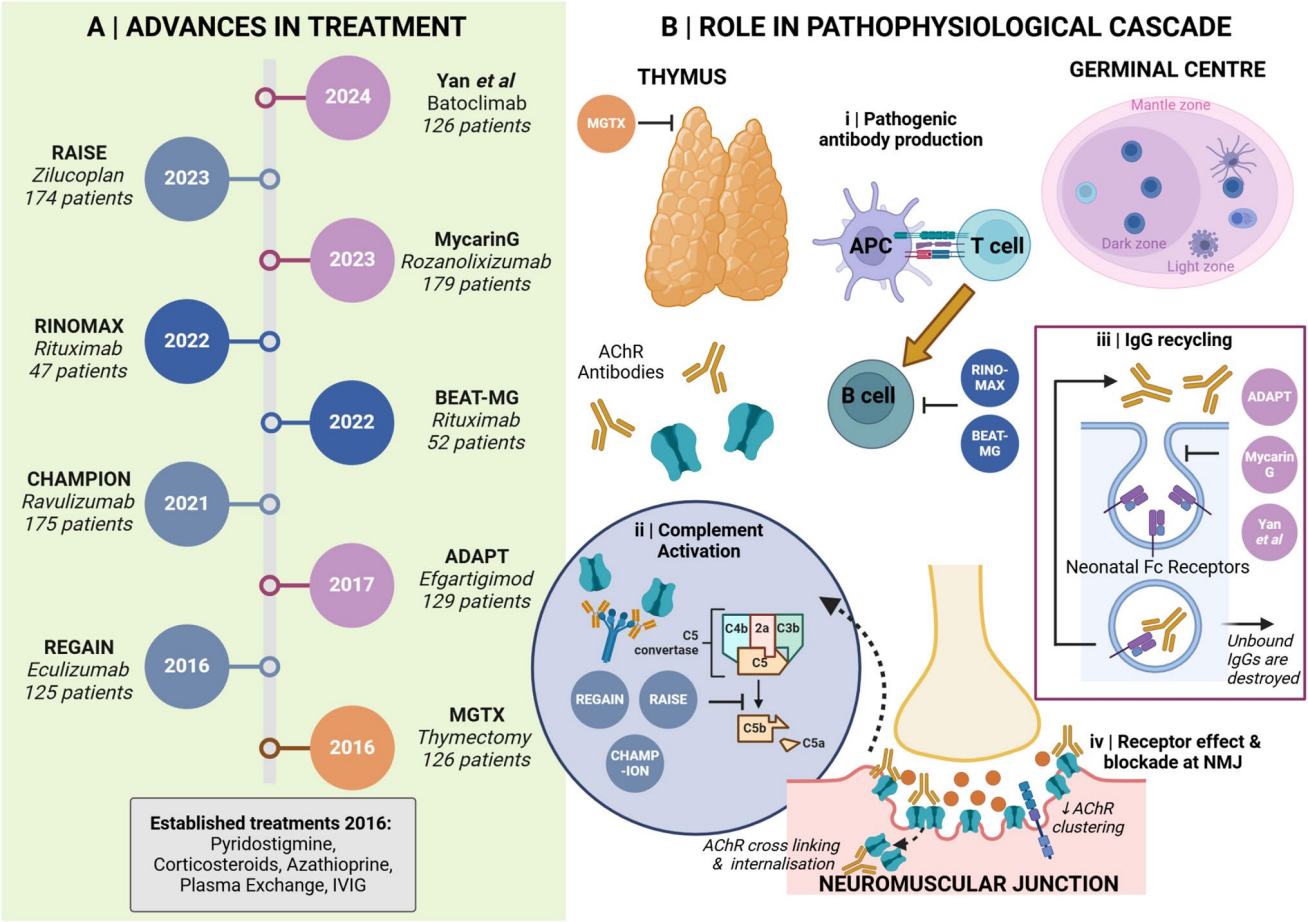
Advances in treatment in acetylcholine-receptor antibody (AChR-Ab)-positive myasthenia gravis (MG). Panel **A** depicts a timeline of new treatments available since 2016. Panel **B** shows the role of these new therapeutic approaches in the pathophysiological cascade of AChR-Ab MG, with the three main mechanisms (complement activation, cross-linking and internalization, and reduced receptor clustering) delineated in (ii) and (iv). (i) Thymectomy acts at the level of the thymus to halt autoimmunization and pathogenic antibody production. Anti-CD20 therapy acts later in this pathway to remove antibody-secreting B cells from circulation; (ii) Trials of anti-complement agents work by disrupting the complement cascade activated by IgG1 antibodies, which, left unchecked, leads to tissue damage at the neuromuscular junction; (iii) the mechanism of FcRN inhibition is by blocking IgG recycling; pathogenic antibodies cannot bind to occupied FcRN receptors and are degraded instead of being returned to the circulation; (iv) at the NMJ, pathogenic antibodies can act by direct receptor blockade as well as by receptor cross-linking and internalization. *Ab* antibody, *AChR* acetylcholine receptor(s), *APC* antigen-presenting cell, *FcRN* neonatal Fc receptor, *MG* Myasthenia Gravis. Image created with Biorender

**Fig. 3:**
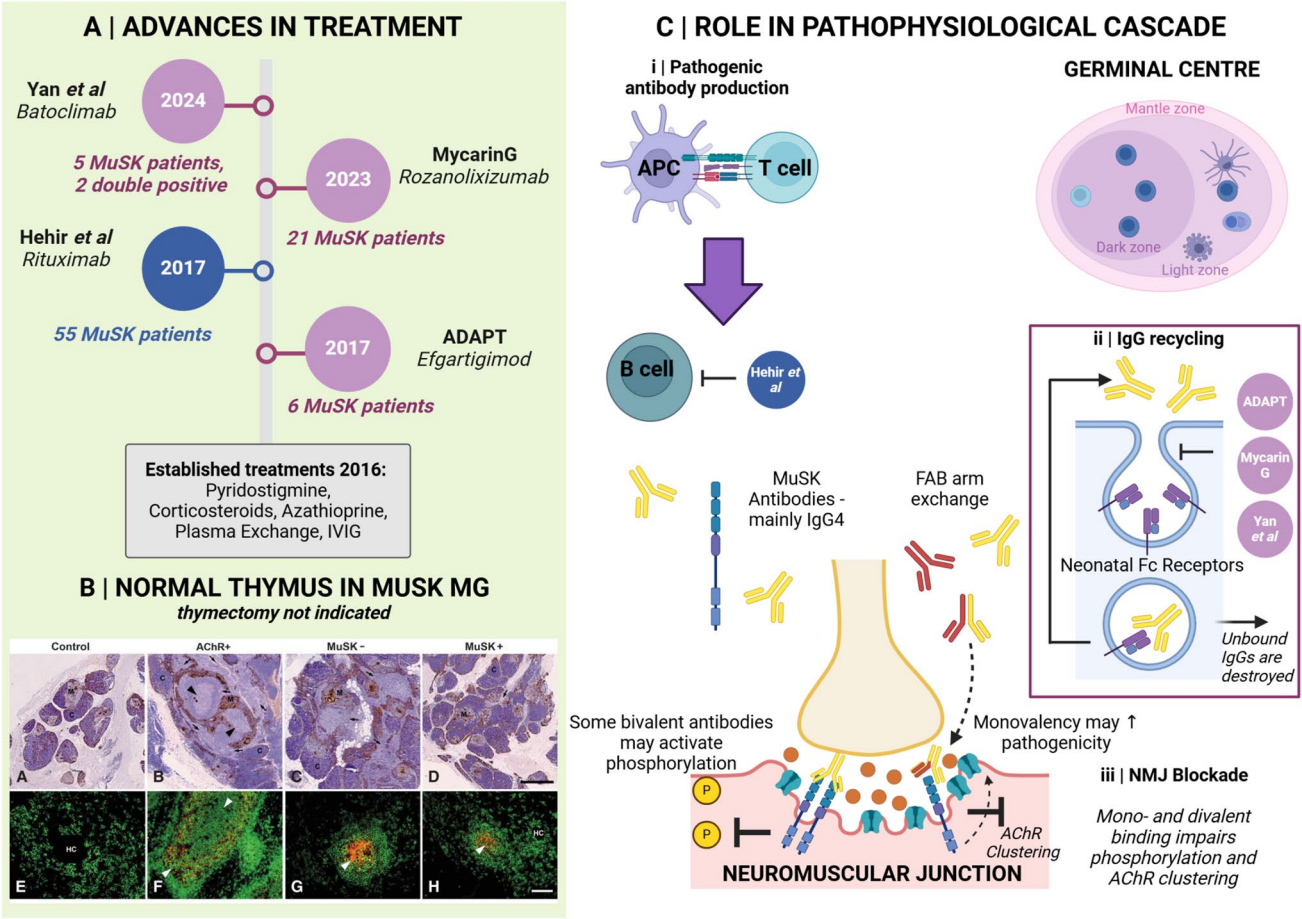
Advances in treatment in muscle-specific kinase antibody (MuSK-Ab) positive myasthenia gravis (MG). Panel **A** depicts a timeline of new treatments available since 2016. Panel **B, A–E** shows example of thymic pathology in control (**A** and **E**), AChR-Ab (**B** and **F**), seronegative, probably low-affinity AChR by modern testing methods (**C** and **G**), and MuSK-Ab (**D** and **H**). Immunofluorescence staining for CD20-expressing (green) and CD35-expressing follicular dendritic cells (red) revealed a lack of CD35 cells in control thymus (**E**), whereas germinal centers were more extensive in AChR-Ab (**F**) than negative **H** patients. Also, germinal centers were only found in 4/14 MuSK thymi examined [24]. Therefore, due to lack of thymic pathology, thymectomy is not indicated in MuSK MG. Panel **C** shows the role of new therapeutic approaches in the pathophysiological cascade of Musk MG. (i) Anti-CD20 therapy removes some antibody-secreting B cells and their precursors from circulation; (ii) the mechanism of FcRN inhibition is by blocking IgG recycling; pathogenic antibodies cannot bind to occupied FcRN receptors and are degraded instead of being returned to the circulation; (iii) most MuSK antibodies are of the IgG4 sub-class and do not activate complement. Their mechanism of action is via receptor blockade, impeding receptor clustering, and alteration of onward phosphorylation mechanisms. *Ab* antibody, *AChR* acetylcholine receptor(s), *APC* antigen-presenting cell, *FcRN* neonatal Fc receptor, *MG* Myasthenia Gravis, MuSK muscle-specific kinase. Image created with Biorender. Panel B reproduced from: Leite MI, Scröbel P, Jones M, et al. (2005). Fewer thymic changes in MuSK antibody-positive than in MuSK antibody-negative MG. Ann Neurol 57:444–448. License number 5760141271853. Copyright © 2005 American Neurological Association)

Treatment goals in MG can be conceptualized as meeting three goals at the pathophysiological level: (1) protecting the neuromuscular junction; (2) removing the effector cell types responsible for producing pathogenic antibodies and/or the antibodies themselves from circulation (Figs. 1–3); and (3) reducing or even halting the process of autoimmunization which underlies the autoimmune attack. At the time of our previous review, most available treatments were accepted through retrospective use and expert opinion, although they could be broadly categorized as belonging to the first two of these groups [19]. However, historically available immunosuppressive options come with notable side effects (corticosteroids and azathioprine), require frequent, inconvenient pulsing (intravenous immunoglobulin (IVIG) and plasma exchange), and may be inadequate to control disease in up to 15% of cases [20, 21].

The advent of thymectomy for AChR-positive MG, as discussed above, addresses disease cascade at the site of autoimmunization since thymic germinal centers foster creation of AChR-antibody producing plasma cells [23–25]. However, the biggest expansion in disease modifying therapies has taken place in the second category, with several landmark trials completed and novel agents available since our previous review.

#### Protecting the NMJ in AChR-antibody MG

The IgG1 antibodies of AChR-antibody MG trigger complement activation, causing tissue damage at the post-synaptic membrane of the neuromuscular junction (NMJ) [26]. Eculizumab, a monoclonal antibody (mAb) which inhibits the C5 component of the complement cascade, was under investigation in 2016 and reported on its phase 3 trial, termed REGAIN, in 2017 (Fig. 2) [27]. This enrolled 125 AChR-antibody-positive patients, 62 assigned to Eculizumab and 63 to placebo. Eligible patients were adults (18 or older) with refractory disease treated with standard immunotherapy for at least 12 months without symptom control. Although the trial did not meet its primary endpoint (overall change from baseline in the MG activities of daily living (MG-ADL) scale between the two groups), several secondary endpoints in a range of validated tools including the myasthenia gravis composite score (MGC), QMG and MG Quality-of-Life 15 (MG-QoL15) did suggest a beneficial impact [27]. Furthermore, the open-label extension phase provided evidence of sustained improvement at 130 weeks of Eculizumab, with 88% achieving Myasthenia Gravis Foundation of America (MGFA) post-intervention status of ‘ improved’ and 57.3% with ‘minimal manifestation’ [28]. Eculizumab was approved by the US Food and Drug Administration (FDA) and European Medicines Agency in 2017 for AChR-positive MG [29, 30] and has since been joined by two further C5 inhibitors (Fig. 2 and Table 1) [31–35]. Serious infections and death have been reported with Eculizumab in neuromyelitis optica spectrum disorder patients on long-term immunosuppression. This group of patients is particularly vulnerable due to severity of disease and length and multiple immunosuppressive agents [36].

**Table 1:** Selected newly-licensed and late-stage investigatory disease modifying therapies in MG

Name / Approvals at-a-glance (2024)	Approval and trial notes	Indication	Description	Mechanism of action
Category: NMJ protection by complement inhibition				
Eculizumab FDA EMA	FDA [29] and EMA (2017) [30]; NICE on hold Phase 3 pediatric trial (NCT03759366) under way	AChR Ab + gMG adults (Refractory in EU)	IgG2/4 kappa humanized mAb with murine CDRs [37]	Blocks C5 cleavage and complement cascade [27]
Ravulizumab FDA EMA	FDA [32] and EMA (2022) [33]; NICE pending Phase 3 pediatric trial (NCT05644561) under way	AChR Ab + gMG adults (‘Add on’ therapy in EU)	Humanized IgG4 mAb [31]	Blocks C5 cleavage and complement cascade [31]
Zilucoplan FDA	FDA (2023) [35] Open label pediatric trial (NCT06055959) planned	AChR Ab + gMG adults	Small (3.5 kDa,15 AA including four unnatural AAs) macrolytic peptide [38]	Blocks C5 cleavage / complement cascade and C5b to C6 binding [34, 38]
Gefurulimab (ALXN1720)	Phase 3 trial (NCT05556096)	AChR Ab + gMG adults	Bispecific miniaturized and humanized nanobody [39]	Blocks C5 cleavage and complement cascade. Binds human albumin which increases its half-life [39, 40]
Pozelimab + Cemdisiran	Phase 3 trial (NCT05070858)	AChR / LRP4 Ab + gMG adults	Stabilized human mAb to C5 with different epitope to Eculizumab/Ravulizumab, and siRNA to C5 [41]	Blocks C5 cleavage, complement cascade and liver synthesis of complement [41, 42]
Category: Remove effector antibodies				
Efgartigimod FDA EMA	FDA (2021) [43], EMA (2022) [44]; NICE due 2024 Phase 3 pediatric trial (NCT05374590) under way	AChR Ab + gMG adults (‘Add on’ therapy in EU)	IgG1 Fc fragment binding to FcRN [45]	Inhibits endogenous IgG recycling via competitive FcRN blockade [45]
Rozanolixizumab FDA	FDA (2023) [46], EMA orphan status, NICE under way	AChR and MuSK Ab + gMG patients	Humanized IgG4 mAb to FcRN [47]	Inhibits endogenous IgG recycling via competitive FcRN blockade and lysosomal pathway inhibition [47, 48]
Nipocalimab	Phase 3 trial (NCT04951622) Phase 3 trial (NCT05265273)	gMG adults gMG children 2–18 years	Fully human aglycosylated IgG1 mAb to endosomal and extracellular FcRN [49]	Inhibits endogenous IgG recycling via dual-compartment FcRN blockade [49]
Batoclimab	Phase 3 trial (NCT05403541)	gMG adults	Fully human IgG1 mAb to FcRN with modifications to reduce cytotoxicity [50, 51]	Inhibits endogenous IgG recycling via competitive FcRN blockade [50, 51]
Category: Remove effector cell types				
Rituximab NICE biosimilar	Not licensed, but biosimilar approved by NICE in the UK in 2021 [52, 53]	gMG especially MuSK Ab +	Chimeric mouse-human anti-CD20 mAb [54, 55]	Depletes range of B cells (illustrated in Fig. 1) [54, 55]
Inebilizumab	Phase 3 trial MINT (NCT04524273)	AChR + MuSK + GMG adults	Humanized IgG1 anti-CD19 mAb [56]	Depletes B cells [56] (Fig. 1, with more activity against longer-lived populations)
Telitacicept	Phase 3 trial (NCT05737160)	gMG adults 18–80	Fusion protein formed from TNFRSF13B and human IgG Fc receptor [57, 58]	Inhibits B cell activators APRIL and BAFF [57, 58]
Satralizumab	Phase 3 trial (NCT04963270) Trial halted March 2024 due to not meeting endpoints	Seropositive gMG 12 + years	Humanized mAb to soluble and membrane bound IL6 receptors [59, 60]	Blocks IL6 signaling and T cell activation of B cells [61]
CAR T cells	Phase 1b/2a study published	Adult gMG 18 +	CAR T cells (autologous) engineered with a receptor against a plasma cell surface molecule, B-cell maturation antigen (BCMA) [62]	Depletion of specific cell populations involved in immunopathological cascade in MG [61]
CAR T cells	Phase 1 study recruiting (NCT05451212)	Adult MuSK positive 18 +	CAR T cells engineered against the MuSK autoantigen [63]	Depletion of MuSK-specific B cells [63]
Category: reduce or halt autoimmunization				
Thymectomy	Case–control study (NCT04158661)	All adults 18 + with MG	Minimally invasive thymectomy	Removal of thymus as site of immunization and autoantibody production

Clinical trials taken from ClinialTrials.gov, supplemented by individual references where indicated. *AA* amino acid, *Ab* antibody, *AChR* acetylcholine receptor, *BAFF* B cell-activating factor, *CAR* chimeric antigen receptor, *CDR* complementarity-determining region, *APRIL* a proliferation-inducing ligand, *EMA* European Medicines Agency, *EU* European Union, *Fc* fragment crystallizable, *FcRN* neonatal Fc receptor, *FDA* US Food and Drug Administration, *gMG* generalized myasthenia gravis, *IgG* immunoglobulin, *LRP4* Low-density lipoprotein receptor-related protein 4, *mAb* monoclonal antibody, *MuSK* muscle-specific kinase, *NICE* National Institute for Health and Clinical Excellence, *RNA* ribonucleic acid, *siRNA* small interfering RNA, *TNFRSF13B* TNF receptor superfamily member 13, *UK* United Kingdom.

Ravulizumab, also a humanized anti-C5 mAb, was the trial drug in the CHAMPION trial in 2022, which also enrolled adult patients. Both the MG-ADL and QMG delineated improved outcomes in the Ravulizumab (*n* = 86) group compared to placebo (*n* = 89) [31]. A key difference is the extended dosing interval (8 weeks once established, compared to every 2 weeks for Eculizumab) which may be relevant to patient preference factors [64]. A third C5 inhibitor, Zilucoplan, a cyclic peptide, was added in 2023 with the RAISE trial [34]. Similar to the mAbs, scores on accredited MG rating scales improved with Zilucoplan, and it has the advantage of being given as a self-administered subcutaneous injection. The safety profile of these agents is acceptable, but meningococcal vaccination for protection against encapsulated organisms is mandatory prior to drug initiation. Phase III extension and Phase IV trials, as well as real-world clinical and safety data collection will be required to determine the long-term safety profile of complement inhibition in MG.

#### Removing culprit antibodies: efficacy in AChR-antibody and the first licensed agent in MuSK-antibody MG

The neonatal FcRN receptor is critical to the recycling of circulating IgG antibodies in vivo (Fig. 2 and 3) [65, 66]. Blocking FcRN receptors results in endogenous immunoglobulins being targeted for lysosomal degradation instead of recycled, credited with achieving a 70% reduction of total circulating IgGs comparable to plasma exchange [66]. Two recently approved therapies make use of this mechanism for therapeutic gain. Efgartigimod (FDA-approved in 2021) is an IgG1 Fc fragment dosed as an intravenous infusion, whose binding affinity to neonatal FcRN receptors exceeds that of endogenous IgGs and thus prevents their return to circulation. In the phase 3 ADAPT trial, its use compared to placebo attained clinically meaningful improvements in the MG-ADL [45].

By comparison, Rozanolixizumab is a subcutaneous formulation and targets the FcRN in the form of a humanized IgG4 mAb, but with a parallel downstream effect of discarding endogenous IgGs from circulation, probably via the lysosomal pathway [47, 48]. In the pivotal MycarinG trial, this drug achieved significant improvements compared to placebo in established outcome MG scales, as well as a new patient-rated outcome measure, the Myasthenia Gravis Symptoms PRO [67], introduced to capture disease impacts more effectively, including recognizing the importance of fatigue [47]. Total IgG levels in both trials were reduced by 60–70%, and in both cases antigen-specific antibody reduction appeared to track total clearance, which was more tightly seen with Rozanolixizumab treatment [45, 47]. The main side effect of this medication class is headache although infections can also occur and may be serious. On-therapy live or live attenuated vaccinations are contra-indicated [45, 47].

Just reported are the phase 3 results of the Batoclimab trial, a humanized IgG1 antibody to the FcRN, administered subcutaneously, which studied 132 patients and also found significant and sustained improvement on the MG-ADL scale (31.3%, 20/64 randomized to placebo versus 58.2%, 39/67 to Batoclimab in the first cycle of treatment). Frequent side effects included peripheral edema, and upper and lower respiratory tract infections [68]. This treatment is not yet approved by the FDA (Table 1).

Both the approved FcRN-inhibition phase 3 trials included MuSK-MG patients (Fig. 3 and Table 1) (six in ADAPT, and 21 in MycarinG) and in 2023 Rozanolixizumab became the first FDA-licensed MuSK-MG agent in addition to being approved for AChR-antibody disease [46]. In fact, MycarinG sub-group analysis suggested MuSK patients had a higher reduction in MG-ADL (reduction of four to seven points, compared to around three points, in the different dosing groups) scores compared to their AChR counterparts (derived from a total of 13 MuSK- and 120 AChR-MG patients receiving active treatment) [47]. The Batoclimab cohort included a very small (two in active and three in placebo group) number of MuSK patients.

#### Removing effector cell types: early is best?

As a mAb to the CD20 marker widely found on B cell populations (Fig. 1), Rituximab is well established in multiple autoimmune diseases and could be anticipated to deplete effector cell types in MG too [52]. Until recently, its use (often in refractory scenarios) was based on observational evidence and case series, some summarized in our previous review, which suggested a promising role particularly in MuSK-MG [69].

Randomized controlled trial (RCT) evidence became available in 2022. The Rinomax anti-CD20 trial recruited new-onset patients, almost all AChR-antibody positive (and none MuSK-antibody positive). The 25 patients in the active arm were more likely to achieve minimal disease manifestation than 22 individuals in the placebo group [54]. By contrast, the BEAT-MG trial, with a similar number of trial participants (52, all AChR-antibody positive, at 5.5 years from disease onset at enrolment), found no meaningful effect in several outcome measures between placebo and treatment groups [55]. This is despite the fact that the Rituximab dose in BEAT-MG, at 375 mg/m^2^ weekly for four weeks, far exceeded the low dose 500 mg infusion given in the early-intervention Rinomax [54, 55].

One possible interpretation is that the Rinomax paradigm, recruiting patients at disease onset, harnessed therapeutic momentum through early removal of effector cells from the circulation, before perpetuation of resistant mechanisms such as complement-mediated NMJ damage and inauguration of CD20-antibody-producing plasma cells in protective niches. Although non-significant, the fall in AChR titers observed in the Rituximab group is in keeping with this interpretation. This might be a lesser risk in the IgG4 predominant MuSK-MG, where complement is not activated, and IgG4-antibody secreting cells including plasmablasts may be preferentially sensitive to CD20 depletion.

While there have been no RCTs of Rituximab in MuSK-MG, in 2017, a multicenter blinded prospective review of 55 patients, a large cohort in such a rare disease, found Rituximab-treated patients were significantly more likely to have a good outcome as assessed by the myasthenia gravis status and treatment intensity (MGSTI) scale (58% (14/24) vs. 16% (5/31)) and less likely to require corticosteroid therapy (29% vs. 74%) and at a lower mean dose (4.5 mg compared to 13 mg daily) [70]. Numerous smaller reports continue to accrue since our 2016 review, continuing to build a meaningful case for Rituximab in MuSK-MG [71].

#### Conclusion: an explosion in therapies in MG

Since our 2016 review, the therapeutic landscape in MG has been considerably enriched, and several late-phase trials are ongoing at the time of writing, including new agents in the category classes described above, emerging agents in categories already established in other autoimmune diseases (e.g. IL6 receptor blockade), and novel approaches including CAR T therapy (discussed in Hope 4) [62]. Depletion of CD19 B cells (Fig. 1) offers potential to target antibody-secreting plasmablasts and even some long-lived plasma cells. Table 1 summarizes some important therapies currently under investigation. Our next section will explore the clinical role of these new therapies and their integration into existing guidelines.

### New treatments: integration into real world practice and updated guidelines

Our 2016 review summarized European and UK best practice guidelines and advice for expectant mothers [19]. Since then, the considerable expansion of available therapies calls for consideration of how these are integrated into clinical practice and national/international best practice guidelines. It is expected that use of new agents will evolve over time, requiring repeated expert body attention and assessment of parameters including safety, cost, de-escalation and combination options.

2020 saw the release of updated International Consensus Guidance, incorporating expert views on thymectomy, Rituximab, and Eculizumab [16]. In summary, advice was for early consideration of thymectomy in those aged 18–50 with nonthymomatous AChR-antibody positive disease, and, reflecting the inclusion of individuals aged up to 65 in the MGTX study, in all patients with AChR-positive MG deemed non-responsive to or intolerant of initial immunotherapy. Early use of Rituximab in MuSK-antibody-positive patients and Eculizumab for severe or refractory AChR-antibody gMG was also recommended. Other complement inhibitors and FcRN inhibitors were not yet available for discussion. In addition, Japanese guidelines published in 2022, including Eculizumab, stated this should be considered when more established modalities of IVIG or plasma exchange are inadequate to control symptoms. These guidelines also highlighted the role of thymectomy in AChR-antibody-positive disease [72].

**Fig. 4:**
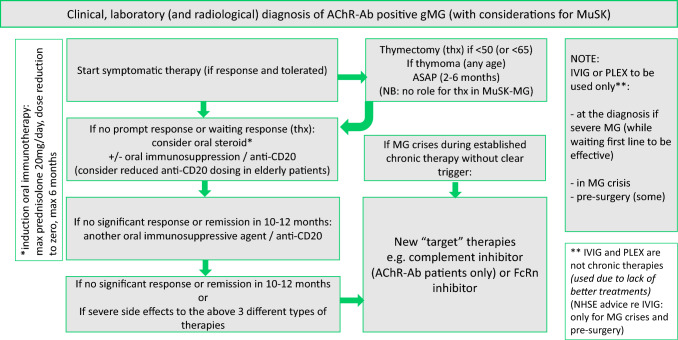
Treatment algorithm for gMG with AChR or MuSK antibodies. After clinical, laboratory (and radiological) diagnosis of gMG, start symptomatic therapy (pyridostigmine). In AChR-antibody positive cases under the age of 65, and all MG patients with thymoma, thymectomy should be considered. Oral steroids (aim for no more than 20 mg prednisolone/day induction dose), other oral agents (e.g. azathioprine/mycophenolate mofetil/ciclosporin/methotrexate), or anti-CD20 can be started if insufficient response to symptomatic medications or while waiting for a thymectomy procedure and subsequent response, which may take up to two years for full therapeutic effect. If severe side effects or an inadequate response to this stepped approach at 10–12 months, consideration should be given to adding in a new targeted therapy such as a complement inhibitor (AChR-Ab patients) or FcRN-inhibition (AChR and MuSK-Ab patients). IVIG and PLEX are not chronic therapies and their use is advised for rescue therapy at disease onset or in MG crisis, and, for some patients pre-surgically. The updated German guidelines propose a comparable approach [73]. *Ab* antibody, *AChR* acetylcholine receptor(s), *FcRN* neonatal Fc receptor, gMG (generalized) Myasthenia Gravis, *IVIG* intravenous immunoglobulin, *MuSK* muscle-specific kinase, *NHSE* National Health Service England, *PLEX* plasma exchange, *thx* thymectomy

The above guidelines situate the use of new agents in refractory disease as do current NHS England recommendations focusing on Rituximab’s use in resistant disease [53]. However, it is increasingly recognized that early assertive treatment is best poised to gain symptom control and achieve the goal of minimal disease manifestation on ≤ 5 mg prednisolone a day, as well as limit permanent NMJ damage, particularly in young patients [72]. Early intervention could provide tangible benefits and rapid stabilization in severe disease, a concept we illustrate in Fig. 4, and in support of which there is some trial evidence. Moreover, the two Rituximab trials described above demonstrate the potential advantage of early treatment before entrenchment of permanent muscle weakness [54, 55]. In our experience, maximum therapy requirements are personalized and tailored to factors including age, occupation, performance status (a measure of physical functioning), life goals, illness beliefs, and disease severity. An important demographic group, elderly patients, are variably represented in the trial landscape: some (MGTX, Zilucoplan) had an upper age limit (65 and 74, respectively) [1, 34]; others did not have an upper age limit but mean ages of patients in their treatment arms reflected a mainly younger demographic in their 40s or 50s [27, 31, 45, 47]. The Rinomax trial was most inclusive of elderly patients, with a mean age in the treatment arm of 67.4 years and a standard deviation of 13.4 years, which represents better the current demographics of patients in cohorts of gMG.

In the pivotal trials of complement inhibition [27, 31, 34], clinical effect, as assessed by the MG-ADL and QMG score, was apparent within one week with all three approved agents (Eculizumab, Ravulizumab, and Zilucoplan). Similarly, FcRN inhibition has been shown to drop total circulating IgG and antigen-specific IgG within a week of initiation of therapy [45, 47], depleting circulating antibodies at a similar rate and proportion to plasma exchange, while removing some risks specific to plasma exchange such as volume shifts, line infections and bleeding [65, 66]. This may be particularly helpful in elderly populations. One aspect of FcRN and complement inhibition is these are likely to be long-term modalities as their mechanisms do not address the cellular root cause [74]. Their role in the hyper-acute setting will remain under exploration; MGFA class II–IV (mild to severe generalized weakness) and stable disease for at least four weeks was a trial entry criterion [31, 34, 45], with certain trials (Eculizumab, Rozanolixizumab) additionally explicitly barring patients in crisis, and Rozanolixizumab specifying a maximum of class IVa (thus excluding patients with predominant bulbar/respiratory symptoms) [27, 47]. Also, it is worth noting that in these emerging therapies, trial protocols allowed concurrent use of existing and rescue standard therapy.

Two new major European guidelines were recently issued at time of writing this review. German guidelines arrived in 2023, focusing on assessment of disease activity through validated tools, and the use of new agents in very active or refractory patients [73]. These were followed in 2024 by new Nordic guidelines, and the main novel points were that Rituximab at single doses not exceeding 500 mg could be used early in disease instead of steroids and azathioprine, that availability of new complement and FcRN inhibitors depended on local factors and should be reserved for difficult-to-treat patients, and the role of thymectomy was emphasized [75]. The UK ABN guidelines, dating from 2015, require a refresh [6]. Updated guidelines will be valuable, but should be viewed as a framework within which individualized decisions can be taken, taking into account patient-level factors, such as disease duration, disease severity, past and current immunosuppressive medications, and co-morbidities. Moreover, the increasing prevalence of LOMG [76] demands careful thought about steroid and immunotherapy dosing in the older patient (Fig. 4). As the number of new therapies rises, close working relationships between general and sub-specialty neurologists will be critical, with tertiary centers able to advise at all MG stages and balancing appropriate referral with support to manage patients locally where possible.

### Biomarkers: toward precision treatment in MG

While a number of exploratory biomarkers are under investigation (Table 2), most remain distant from clinical use. For example, despite significant research activity and some emerging profiles (Table 2), circulating miRNAs would still require multicenter laboratory standardization and studies to gain credence [77]. The biomarkers most studied and proximal to clinical utility are the circulating immune cell repertoire and the humoral response itself. Indeed, B cell subsets are routinely measured in clinical practice in conjunction with Rituximab use [53], and IgG levels and immune cell characterization are incorporated in recent[34, 45, 47, 54, 55] and in-progress trial designs. Genetic polymorphisms may predict response to some therapies, for example, fragment c gamma receptor 3A (FCGR3A) polymorphisms are established as impacting on-Rituximab relapses and a re-dosing requirement in Korean patients with neuromyelitis optica spectrum disorder [78]. Similarly, a mis-sense mutation in C5 (c.2654G > A, conferring a p.Arg885His polymorphism), was found to abolish Eculizumab binding and underlie poor response to Eculizumab in Japanese and Chinese patients [79].

**Table 2:** Exploratory biomarkers in MG

Biomarker	Potential utility & MG subtype	Summary of findings
miRNAs	Diagnosis of OMG vs. gMG	• Phase 3 trial enrolment (NCT05888558) under way
	Diagnosis of MuSK + MG [80]	• Increased serum let-7 miR-let-7a-5p, miR-let-7f-5p, miR-423-5p and miR-151a-3p levels in MuSK-MG cohort compared to HCs [80]
	Treatment response in AChR Ab + MG [81–84]	• Increased serum miR-150-5p and miR-21-5p titers in MG compared to HCs • Decreased serum miR-150-5p titers post-thymectomy, and decreased serum exosomal miR-150-5p in parallel with improved clinical status after Rituximab treatment • Decreased serum miR-150-5p and miR-21-5p titers in an immunosuppressed MG group compared to non-immunosuppressed MG group, but no association with clinical status • Serum miR-323b-3p, -409-3p, and -485-3p titers significantly decreased in immunosuppression non-responsive versus immunosuppression responsive group, and serum miR-181d-5p and -340-3p titers significantly increased in immunosuppression the non-responsive group
	Disease progression and treatment response in LOMG [85]	• Serum miR-150-5p, miR-21-5p and miR-30e-5p titers negatively correlated with MGC score after immunosuppression
	Differentiation between OMG and SGMG [86]	• Serum miR-30e-5p is highly sensitive in differentiating OMG and secondarily generalized gMG
Heat shock proteins	HSC 71: Disease progression and treatment response in gMG [87]	• Serum anti-heat shock cognate protein 71 antibody (HSC71 Ab) titers significantly elevated in gMG compared to HCs • Serum HSC71 Ab titers significantly decreased in parallel with improved clinical status • In patients refractory to acetylcholinesterase inhibitor treatment, the initiation of tacrolimus therapy was associated with improved clinical scores and reduced HSC71 Ab titers
	HSP90α: Treatment response in thymomatous and non-thymomatous MG [88]	• Serum titers of HSP90α significantly increased in patients with thymomatous and non-thymomatous MG compared to healthy controls • In thymoma patients, high serum HSP90α titer associated with increased rate of tumor recurrence • Complete tumor resection correlated with decreased serum HSP90α titers • Non-thymomatous MG patients who were thymectomy non-responsive had significantly increased preoperative HSP90α serum concentrations compared to thymectomy responsive patients
	HSP 70: MG diagnosis [89]	• Anti-Heat Shock Protein 70 (Hsp70) antibody titers increased in MG and Guillain-Barré syndrome compared to HCs and MS patients
Neurofilament light chain	Disease progression in adults 18+ with MG [90]	• Serum neurofilament light-chain titers elevated in MG cohort compared to HC • No statistical association between serum neurofilament light chain titers and clinical status (QMG and MG-ADL scores)
Gut markers	Disease susceptibility, comparison to healthy controls, and possible therapeutic targets [91–95]	• Several studies depicting altered fecal microbiota, reduced diversity, and dysbiosis in MG vs. controls. Specific relationships include putative etiological roles for *Lachnoclostridium* and *Faecalibacterium* and interventional targets of *Bacteroidetes* and *Desulfovibrionaceae* • In one study, serum titers of systemic inflammatory markers were elevated in MG, correlated to gut dysbiosis, and MG patients had increased carriage of certain species including *Streptococcus*
	Diagnosis and disease progression in all MG subtypes [96]	• Panel of microbial and metabolic biomarkers identified from stool samples were capable of discriminating MG and HCs with 100% accuracy • Several indexes of microbial diversity negatively correlated different with increased QMG scores when patients were divided into disease severity groups
	Diagnosis of OMG and gMG [97]	• Panel of microbial and metabolic biomarkers identified from stool samples were capable of discriminating OMG, gMG and HCs
Single-fiber EMG	Treatment response in seronegative OMG [98]	• Increased orbicularis oculi SFEMG jitter in patients had a high predictive value for therapeutic response
	Disease progression in mild AChR+ MG [99]	• Increased jitter and increased blocking were associated with disease exacerbations
Smartphone data	Disease status and exacerbation in adults 18+ with MG [100]	• Pilot study to phenotype MG patients and gather digital markers of impending exacerbation via smartphone data collection

Clinical trials taken from ClinialTrials.gov, supplemented by individual references where indicated. *Ab* antibody, *AChR* acetylcholine receptor *gMG* generalized myasthenia gravis, *HCs* healthy controls, *HSC71* heat shock cognate protein 71, *HSP 70* heat shock protein 70, *HSP90α* heat shock protein 90α, *LOMG* late-onset myasthenia gravis, *MG* myasthenia gravis, *MG-ADL* myasthenia gravis activities of daily living scale, *MGC* myasthenia gravis composite scale, *miRNA* microRNA, *MuSK* muscle-specific kinase, *OMG* ocular myasthenia gravis, *QMG* quantitative myasthenia gravis score, *SGMG* secondarily generalized myasthenia gravis

#### Progress and a clearer picture in MuSK-MG

At the time of our earlier review, MuSK antibodies were already considered to be reflective of disease activity [101]. Antibody titers were positively correlated with disease severity scales and shown to fall after immunotherapy in individual patients [101]. Titers tended to decrease with time and some patients became seronegative [101]. Since then, further reports have delineated a rise in MuSK-antibodies preceding relapse [102, 103], but more comprehensive studies would be valuable [104]. Detailed screening and epitope mapping of anti-MuSK antibodies has extended understanding by delineating, specifically, a relationship between antibodies to MuSK’s IgG-like1 domain and disease severity [105]. This speaks to the underlying pathophysiological mechanisms since this domain is essential to MuSK’s interaction with LRP4, and downstream clustering of AChRs [105]. Pathogenic mAbs to the IgG-like 2 domain have also been implicated, in smaller cohorts [106]. Some investigators have found a sub-population of MuSK antibodies which, contrary to expectations, activate MuSK phosphorylation and a degree of AChR clustering [107–109]. It may be that these bivalent antibodies are non-pathogenic, gaining pathogenic potential only after FAB arm exchange, a property unique to IgG4 sub-class antibodies, and functional monovalency [108]. While the majority of MuSK-MG antibodies are of the IgG4 sub-class, the role of IgG1-3 subclasses is beginning to be recognized [110]; comparable to IgG4 entities, they inhibit AChR clustering, potentially at even greater potency, but via a different, non-canonical pathway, and may activate complement [106].

In tandem, intensive work has been undertaken to characterize specific B cell phenotypes and clones which may be instrumental in, and herald, relapse. This is now supported by several strands of evidence. Antigen-specific IgG4 B cell clones, moreover targeting MuSK’s IgG-like 1 domain, emerged in advance of clinical worsening in MuSK-MG patients treated with bone marrow transplantation [103]. Rituximab-resistant CD38+ and CD27+ plasmablasts and CD20 low B cell clones survive immunotherapy and reconstitute proximal to and mediate MuSK-MG relapse [111, 112]. Ultimately, resistant cells could act as both biomarkers and therapeutic targets for precision-engineered CAR T cells [63]. Since CD19 is found on a wider phenotype of B cells (Fig. 1), anti-CD19 therapy, now in use in neuromyelitis spectrum disorder, may offer the chance to deplete resistant cells [56].

#### Strength and diversity in numbers: a complex picture in AChR-MG

The picture is different with AChRs. Overall evidence at a cohort level suggests quantitative antibody titers less well relate to disease activity[104] although recently, it was shown that intra-individual changes may be of personal disease-monitoring benefit and merits further study [113]. New technology is now delineating in more granular detail what had previously been established of mechanistic heterogeneity between and within individuals, and even within the same antibody clone, with the mainly IgG1 entities able to activate complement, internalize AChRs, and cause receptor blockade [114]. Moreover, it has been shown that complement activation is enhanced by multiple antibodies in concert, targeting different AChR epitopes [115]. These observations highlight why quality, not just quantity, of antibodies matter in determining pathogenicity, and suggest that polytherapy could be needed to curtail a variety of pathophysiological actions at the molecular level. Nevertheless, AChR-Abs were shown to decrease after Rituximab treatment although levels did not predict relapse [116]. Also, more recently, the persistence of thymic-derived B cell clones post thymectomy indicated a poorer response and in some patients paralleled a persistence of AChR-Ab titers [117].

Due to its long-acknowledged role in AChR-MG, interest has naturally surrounded elements of the complement cascade, and the advent of C5-inhibiting therapies makes this even more pressing [26]. Cleaved components of the classical and alternative complement pathways are more abundant in samples from newly diagnosed AChR-MG patients compared to healthy controls [118]. The components were not diminished after established, non-complement focused immunotherapy [118]. While one functional assay of complement did not differ between AChR-MG patients and controls [119], a novel assay capturing membrane attack complex (MAC) activity shows promise as a biomarker of disease activity and response to anti-complement therapy [120]. Similarly, IL6 is under investigation for biomarker potential, and blockade of its receptor evaluated in MG in a phase 3 Satralizumab (IL6 receptor mAb) trial although at time of writing, this trial had been halted (Table 1). In a cross-sectional study, IL6 was found to be elevated in 93 AChR-MG patients compared to age-matched disease controls, and to correlate, albeit weakly, with MGFA status [121]. The benefits of Tocilizumab, another anti-IL6 receptor mAb, have been reported in case reports and observational studies [122–125]. Notably, IL6 and the soluble IL6 receptor are among a detailed biomarker panel trial announced in another autoimmune disease treated with IL6 receptor inhibition, neuromyelitis optica spectrum disorder [126].

#### Don’t forget about T cells

CD4 + T cells are critical partners in many B cell processes relevant to autoimmunity, including class switching, somatic hypermutation, and maturation [127]. It should be remembered the ‘T’ in T cells represents the thymus, an organ critical to their development and education. Populations of two phenotypes of CD4+ T cells, Th_CD103_ (a TNFα secreting population), and Th_GM_, which produce granulocyte–monocyte colony-stimulating factor (GM-CSF) and are pro-inflammatory [128], were found to be sequestered in the thymus and reduced in the peripheral circulation of MG patients, the latter correlating inversely with disease severity [129]. Thymectomy appeared to release these cells back into the peripheral circulation and Th_CD103_ was proposed as a disease biomarker [129].

Another study concluded that the subset of CD4+ T cells consisting of IL17-positive T follicular helper (Th17+) cells dropped more sensitively than plasmablasts post-immunotherapy, and that a greater proportion of Th17+ cells was associated with higher QMG score [130]. This T cell cytokine, among others including an uplift in GM-CSF, receives support from the results of other investigators in MG [131], and could become a marker of MG crisis and longitudinal disease activity [132].

#### Conclusion: guidelines and biomarkers react to new therapies

Guidelines are beginning to incorporate the wealth of new treatments, and biomarkers could further help refine clinical practice in MG. Response to treatment and biomarkers differs between AChR- and MuSK-MG, reflecting the different disease mechanisms (Figs. 2 and 3) and IgG sub-class predominance. Other factors influential to determine clinical practice include both ‘age’ and ‘stage’ of MG–a concept we will develop below.

### Myasthenia gravis: not just ‘age’ but also ‘stage’

**Fig. 5:**
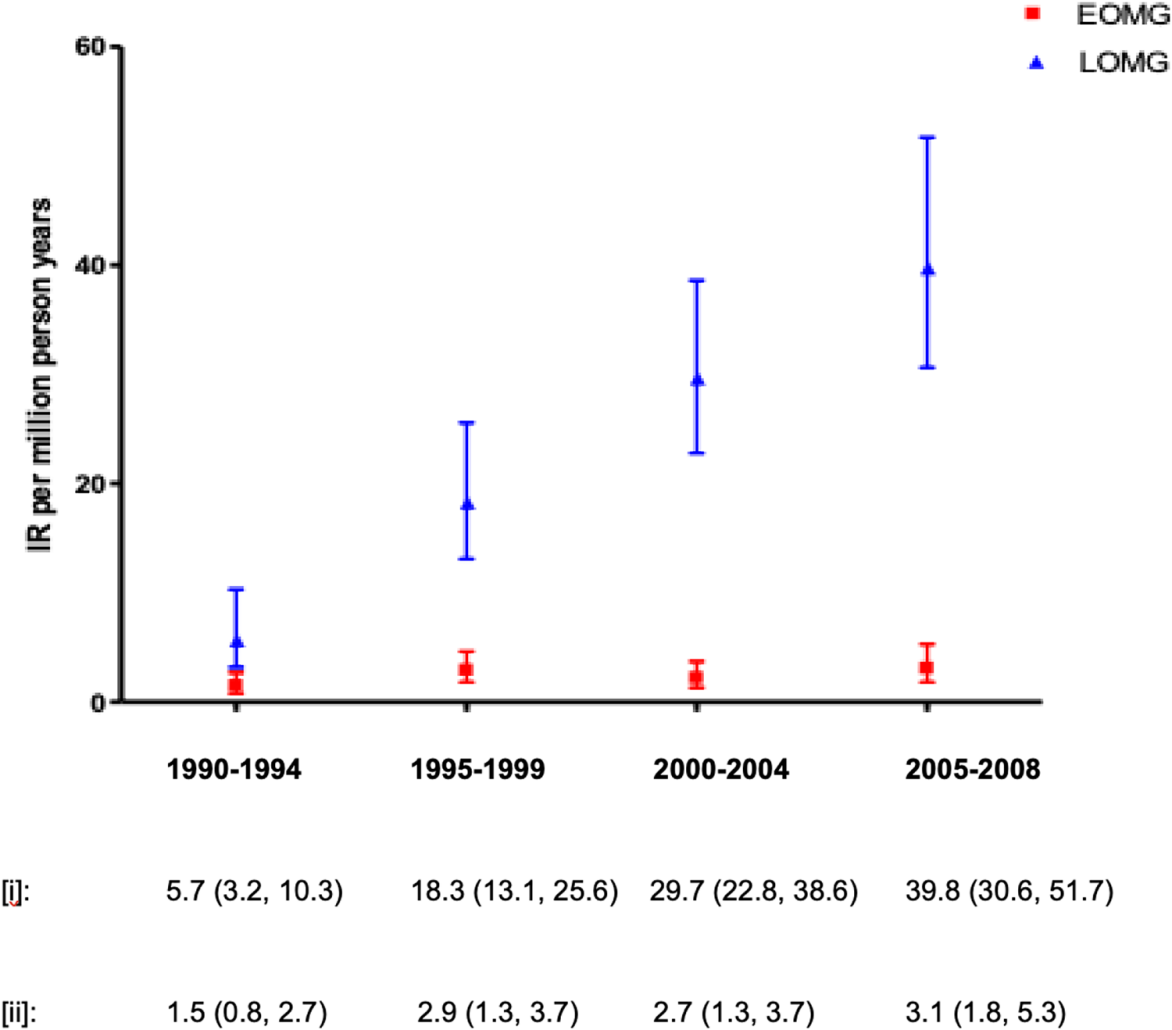
Changing onset of Late-Onset Myasthenia Gravis (LOMG) and Early-Onset Myasthenia Gravis (EOMG) in Northern Ireland from 1990 to 2008. IR is shown in cases per million person-years, error bars represent 95% CI. There is an almost sevenfold increase in IR of LOMG, and although there is a two-fold increase in absolute EOMG IR, the CIs overlap. Reproduced with permission from: AS Carr. Actual world epidemiology of Myasthenia Gravis (Chapter 2). In Mineo TC, editor. *Novel Challenges in Myasthenia Gravis.* Nova Science Publishers, Inc.: 2015. *CI* confidence interval, *IR* incidence rate

Our prior review outlined the increasing incidence of LOMG, an epidemiological phenomenon which has continued to be observed and discussed. In a strictly designed prospective regional UK epidemiological study covering the years 2014–2018, incidence of new MG was at its highest in the > 65s (51.5/1,000,000 for men and 51.3/1,000,000 for women, compared to 17.6/1,000,000 in the population at large) [76]. Also, the > 65 age group was the only group in which incidence rose during the period under investigation [76]. These findings have been echoed in studies from Northern Ireland (Fig. 5), [133] Japan [134], and Germany [135]. Moreover, prevalence doubled in Japan from 2006 to 2017 and in the German study, was the highest in those aged 80 and above [134, 135]. While LOMG will be captured within older cohorts, it is also important to remember middle-aged and above age brackets in prevalence studies also reflect those with EOMG and longstanding disease, especially since the advent of intensive care, as a result of which survival rates of MG have improved [136, 137] and some, [137, 138] but not all recent studies [135, 139], show no increased mortality compared to the general population. There are varying accounts as to whether disease course and likelihood of remission is less favorable in EOMG or LOMG, with studies reporting both comparable [140–143] and worse [144–146] prognoses in older patients. Longitudinal data may provide a synthesis, delineating that, although there may be a more severe-onset in older-onset MG, it subsequently is highly responsive to treatment [137].

As to stages of disease, both historical [136, 147, 148] and more recent cohorts [137, 149] point to an illness course which is usually at its nadir with most crises in the first few years; within two to three years in studies including pre-twenty-first century presentations [136, 147–149], whereas after the turn of the century, this severe stage may be compressed into a single year albeit with most therapeutic benefit achieved in the first two years [137, 149]. These known disease characteristics should be applied to treatment principles of newly diagnosed patients in the current era, especially with the fresh availability and future pipeline of immunotherapies. Even in modern cohorts, 5–20% of patients remain refractory after the initial explosive disease phase and continue to experience exacerbations, and have a high rate of co-morbidities contributing to hospital stays [137, 149]. Presence of thymoma, seronegative status, and co-morbidities may be associated with refractory status although co-morbidities may be as much a result of drug regimens as a reason for non-responsive disease [137, 149]. New, digital technology offers the chance of fresh insights in large cohorts on the longitudinal evolution and burden of disease, for example, fatigue, occupational history, quality of life and caregiver burden [150].

The topic of co-morbidities in all patients and the immunotherapy in older people are emerging as hot topics in the context of changing lifestyles and population demographics. Co-morbidities are prevalent at similar, high, rates in Western populations [141, 151] and in early- and late-onset myasthenia once matched for age [151]. Commonly encountered co-morbidities include hypertension, high cholesterol, diabetes, cataracts, and prostate issues. Multiple co-morbidities and polypharmacy for co-existing conditions are common [141, 151]. These co-morbidities need careful management alongside myasthenia treatment, including immunotherapy. For example, a Danish case–control study did not identify increased risk of major osteoporotic fracture among MG patients on steroid therapy, which was ascribed to adequate bone protection therapy [152]. Increased treatment-related side effects, including fatal infection, have been reported with immunotherapy in older populations [153], but conversely poorer outcomes in older people have been linked to reluctance to initiate more aggressive immunotherapy [142]. A case series of seven LOMG patients aged 55 and above showed encouraging efficacy and tolerability of Rituximab, and advocated its potential use earlier in older patients [154]. Gentle maintenance therapy may harbor a preferential side effect profile to high-dose induction therapy in this age group [155].

#### Conclusion: age and stage are both relevant in myasthenia management

We close the ‘update’ section of our review with a summary of the epidemiology and emerging concepts of immunotherapy in older patients. The advancement of evidence-based treatment protocols for this group represents one of our ‘four hopes for the future’ focusing on improving diagnosis, prognostication and treatment in the myasthenia community, which we now briefly outline below.

## Hope 1: Progress in laboratory testing for rapid and more sensitive serological testing

### AChR: from radioimmuno- to cell-based assays

The neurotoxin α-bungarotoxin isolated from a venomous elapid snake, the Taiwanese many banded krait (*Bungarus multicinctus*), was central to the development of AChR autoantibody assays [156]. It binds the adult and fetal isoforms of the nicotinic acetylcholine receptor with high affinity (nM to pM) and, when labelled with ^125^I, creates a specific, stable, quantifiable target for radioimmunoprecipitation assays [156]. This assay has been used for decades in routine immunology diagnostic laboratories.

**Fig. 6:**
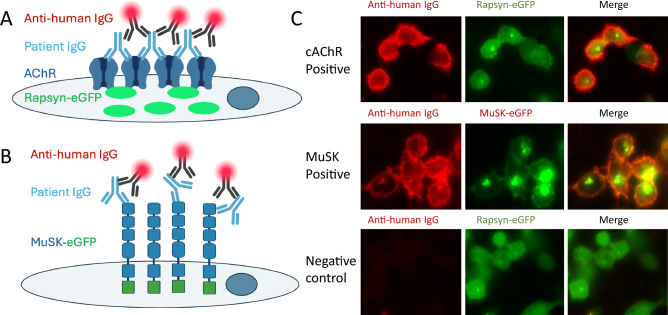
Cell-based assays in myasthenia gravis. Live cell-based assays are the most sensitive method to detect antibodies in people with autoimmune myasthenia gravis. **A** HEK cells transfected with α-, β- and δ-AChR subunits with either the ε or γ subunit for adult or fetal AChR subunits and eGFP-tagged rapsyn is the substrate for the clustered AChR antibody assay. The AChR are shown in blue, the patient antibody in pale blue, the secondary antibody is black with a fluorochrome depicted in red and Rapsyn-eGFP in green. **B** HEK cells transfected with MuSK c-terminally tagged with eGFP forms the substrate for the MuSK live cell-based assay. **C** Examples of an AChR antibody-positive test result in the first row, a MuSK-positive test result in the second row and a negative control for the AChR antibody assay in the bottom row. *AChR* acetylcholine receptor(s), *EGFP* enhanced green fluorescent protein, *HEK* human embryonic kidney, *IgG* immunoglobulin G, *MuSK* muscle-specific kinase. Figure components A and B created with Biorender

Enzyme-linked immunosorbent assay (ELISA) tests for AChR antibody detection generated over the intervening 40 years have not improved on the radioimmunoassay (RIA) test accuracy. However, a proportion of people with generalized MG with identical clinical and electromyographic features to AChR seropositive patients remained AChR antibody-seronegative. The resolution of this discordance was an improved test substrate. Transient transfection of HEK293T cells with AChR subunits in addition to the AChR clustering molecule rapsyn provided a test substrate most reflective of the antibody target in vivo [157]. This clustered AChR cell-based assay uniquely identified a proportion of ‘seronegative MG patients’ as seropositive. Here the human AChRs (adult or fetal) are overexpressed in living cells in the presence of fluorescent-labelled rapsyn that clusters the AChR into punctae. Patient sera are incubated with the cells at room temperature for an hour and AChR-bound antibody is identified using a fluorescent secondary antibody [157]. In three head-to-head studies, this live cell-based assay (CBA) identified 50 new seropositive cases, 18–38% of the patients in these cohorts, who were defined as antibody seronegative by RIA [158–160]. This is now considered the gold standard for the detection of AChR autoantibodies (Fig. 6).

Commercial CBAs generated in a similar fashion to the live CBA, where the substrate is fixed to allow a longer shelf-life of the test substrate, are not as accurate as the live test [164, 165], but the addition of a fluorescence amplification step improved the sensitivity of a commercial fixed CBA from China by 12% over RIA or ELISA [161]. Despite the improved sensitivity, a small proportion of MG sera found negative by fixed CBA was positive by RIA (34/1512 (2%) or ELISA (33/1511 (2%)). Similar studies with live CBAs are needed, but RIA has been superseded by cell-based assays for the detection of AChR-IgG.

### Testing times in MuSK

MuSK antibodies were first identified binding to COS7 cells transiently transfected with rat MuSK and by ELISA on purified rat MuSK extracellular domains [162]. A specific, commercial radioimmunoassay was then developed using the extracellular domains of either rat or human MuSK [163, 164]. A decade later saw the beginning of in-house and commercial live and fixed CBAs for MuSK antibody detection.

A few head-to-head studies suggest the MuSK RIA, ELISA and commercial fixed CBA appear equivalent, but not 100% concordant, with a few missed cases on each test across studies [165–167]. An in-house fixed CBA appeared marginally superior to RIA and ELISA in one large study where 2043 MG patients were screened [161]. But the lack of concordance between these three test systems remains. Of 63 MuSK-positive individuals, 47 were concordant, the in-house fixed CBA identified a further 13 unique positives while the RIA identified 4 unique samples. In a similar vein, not all live assays are equivalent. When Hep-2 M4 cells were stably transduced with human MuSK, only 25/34 RIA-positive samples were identified as positive [168]. However, the choice of cell and the method of expression may make a difference. When the test substrate was HEK cells transiently transfected with full-length human MuSK in six studies, an additional 32 MuSK-positive patients were identified [160, 169–172]. There were 136 RIA-positives in these studies. Hence, a 24% increase in sensitivity for MuSK antibodies. A lack of controls precludes an examination of test specificity.

### The future of laboratory testing in MG

These data show that the initial RIAs for AChR and MuSK antibody detection have been superseded by live CBAs. Live CBAs are time-consuming and currently the remit of specialist laboratories. They require streamlining before being brought into routine clinical laboratories.

## Hope 2: Effective protection of the NMJ

Similar to the concept ‘time is brain’ [173], we propose here the concept of timely NMJ protection. As illustrated in Fig. 4, following clinical, laboratory, and, where appropriate, radiological diagnosis of MG, initiation of prompt and effective treatment is essential to safeguard NMJ function. This strategy is advanced in the context of renewed attention to the chronic atrophy and fatty infiltration that may develop with long-term NMJ pathology [174, 175]. Moreover, it is likely that there is superadded contribution of age-related change at the NMJ including denervation, structural degradation, reduced receptor number, distribution and caliber, [176, 177] of particular consideration in the ageing patient and in LOMG. Therefore, early and effective treatment is needed to preserve structural and biochemical NMJ integrity.

In the immediate post-diagnosis period, until long-term treatment strategy is optimized, IVIG and plasma exchange may be therapeutic options to fend off refractory, burnt out disease (Fig. 4 and example case in Box 1). It is possible that new agents coming on board may also have a role as ‘bridging therapies’ in this context, and investigations into this indication could be worthwhile [178]. The progression of chronic disease over decades, and denervation, in the absence of protective strategems, as captured by historical pathological reports, heralds irrecoverable muscle atrophy and neurogenic degeneration, particularly affecting the bulbar musculature, which retrospective immune therapy cannot repair [179]. This is seen clinically and has been historically evidenced although not investigated with more modern techniques [179].

## Hope 3: Fine-tuning our treatment approach in older populations

We need more knowledge and clinical trials specifically targeted to studying efficacy and tolerability of immunotherapies in older people. Open questions include which of the many new immunotherapies should optimally be used, appropriate dosing, and side effects in this age group. While pilot data have proposed the use of Rituximab in older people with MG, even suggesting it could have fewer risks and reduced cost attached compared to conventional modalities of IVIG and PLEX, it is recognized that more evidence is needed. [154] It should be noted that increased rate and severity of infection have been reported in studies of older (> 75 years) individuals dosed with Rituximab for rheumatoid arthritis at doses of > 2.5 g/year [180, 181]. This may also be the case in older people with MS, alongside co-morbidities and previous severe infection [182]. Studies addressing dosing regimens in adults above retirement age are needed as has been done in vasculitis [155]. In our center, we have had good experience using very low-dose Rituximab in older MG patients without an extensive pre-history of immunotherapies using 500 mg or even 200 mg single doses and monitoring B cell subsets post-treatment (M Isabel Leite, personal observations).

## Hope 4: The power of T cells: a cure for MG?

CAR T therapy harnesses the power of T cells to eliminate specific cells expressing epitopes of antigenic targets. The basic approach is to infuse patients with autologous T cells which have been bioengineered to bear receptors against a disease-relevant entity. It is established in hematological malignancy and considered promising in autoimmune disease, owing to its capability of precise targeting and penetration of immune niches [61, 183].

The largest study to date enrolled 14 patients with gMG (with AChR or MuSK antibodies, one seronegative individual) who received Descartes-08, active against the B cell maturation antigen (BCMA) molecule found on plasma cells [62]. This was an early phase trial, not powered to examine treatment efficacy. Overall, Descartes-08 was well-tolerated with one serious treatment-related side effect of urticaria, and promising metrics on disease trajectory. No hypogammaglobulinemia was observed [62]. Two small-scale clinical reports have reported use of a CD19-targetted CAR T in refractory Lambert–Eaton Myasthenia (LEMS), and a single case report in AChR + gMG [184], with good response [185, 186]. Based on preclinical data [63], a phase 1 trial of MuSK-specific CAR T (MuSK-CAART) is currently recruiting (NCT05451212), representing a further precision medicine step. CAR T methods are likely to expand in the coming years, but open questions include cost, and potential emergence of side effects when used at scale.

## Postscript: Can we predict an aggressive or treatment-responsive disease course?

Of direct relevance when considering high-impact, but also high-effort and high-cost approaches such as CAR T, it would naturally be of high clinical relevance and utility to be able to predict, in advance, which patients will develop aggressive disease and be able to achieve a precision selection of rational, individually tailored therapies. Although in their infancy, many exploratory biological biomarkers (Table 2) await translation into clinically relevant tools. Another promising and potentially highly responsive avenue is the use of data acquired via patient smartphones [100]. A pilot study recently was able to quantify degree of ptosis via patient smartphone ‘selfies’ [187]. Rather than single entities, it is probable that a combination of informative biomarkers could be harnessed for prognostication as was recently demonstrated in another neurological disease, multiple sclerosis [188]. Machine learning approaches are entering into the MG space [189] and, although currently in their infancy, could yield applicable predictive algorithms in future.

Box 1: An illustrative case of AChR-positive gMG, in which chronic IVIG achieved neuromuscular junction protection until the availability of more definitive therapy.

**Table:** 

Patient: Female, 53 years old
• Onset age: 25; thymectomy at 28 (hyperplasia)
• Incomplete response to prolonged and ongoing steroid therapy, and several immunosuppressive agents tried sequentially over the years
• Significant side effects of steroid therapy, including weight gain, early osteoporosis,
• early cataracts and skin changes
• **On chronic monthly IVIG for more than 20 years (until 2021)**
• Dependent on suboptimal response to long-term IVIG, in addition to ongoing steroid therapy. Unable to work
• Since going onto treatment with a complement inhibitor (2021), the patient has improved progressively and regained sustained muscle strength
• Has no clinical manifestations of MG for 3 years and is off other MG treatments for 2 years. Returned to normal life, travelling, busy with family activities and plans to re-start working

## Data Availability

A data availability statement is not appropriate as this is a review article and does not contain original data.
